# Pathogenesis and treatment of osteoporosis in patients with hemophilia

**DOI:** 10.1007/s11657-022-01203-9

**Published:** 2023-01-04

**Authors:** Xiaoyun Lin, Peng Gao, Qian Zhang, Yan Jiang, Ou Wang, Weibo Xia, Mei Li

**Affiliations:** 1 Department of Endocrinology, National Health Commission Key Laboratory of Endocrinology, Peking Union Medical College Hospital, Chinese Academy of Medical Sciences & Peking Union Medical College, Beijing, China; 2 Department of Orthopedic Surgery, Peking Union Medical College Hospital, Chinese Academy of Medical Sciences & Peking Union Medical College, Beijing, China

**Keywords:** Hemophilia, Osteoporosis, Factor VIII, Thrombin

## Abstract

**Introduction:**

Hemophilia is a rare X-linked recessive inherited bleeding disorder caused by mutations of the genes encoding coagulation factor VIII (FVIII) or IX (FIX). Patients with hemophilia (PWH) often have a high risk of osteoporosis and fractures that is usually ignored. Herein, we review the underlying mechanisms of osteoporosis and the increased risk of fractures and their treatment in patients with FVIII or FIX deficiency.

**Methods:**

The PubMed, Web of Science, Embase, and Cochrane Library databases were searched to identify original research articles, meta-analyses, and scientific reviews on the mechanisms or treatment of osteoporosis in PWH.

**Results:**

The pathogenic mechanisms of osteoporosis in PWH are multifactorial and remain unclear. The available evidence shows that FVIII and FIX deficiency may directly affect bone metabolism by interfering with the RANK/RANKL/OPG pathway. Other potential mechanisms of osteoporosis in PWH include thrombin deficiency and the unloading and immobilization of bone, which will affect osteoblast and osteoclast activity by changing the cytokine profiles. The treatment of osteoporosis in PWH includes antiresorptive, anabolic, and dual-action drugs; weight-bearing exercise; fall prevention; and prophylactic coagulation factor replacement therapy. However, clinical studies of the efficacy of anti-osteoporotic agents in osteoporosis of PWH are urgently needed.

**Conclusion:**

This review summarizes recent progress in research on the pathogenesis of osteoporosis in PWH and provides insights into potential treatment for osteoporosis in PWH.

## Introduction

Hemophilia is a rare X-linked recessive inherited bleeding disorder caused by mutations of the genes encoding coagulation factor VIII (FVIII) (hemophilia A) or IX (FIX) (hemophilia B) [1]. Hemophilia A is the most prevalent form of hemophilia, accounting for 85% of patients with hemophilia (PWH), with a prevalence of approximately 1:5000 in male neonates [2]. Hemophilia B accounts for 15% of PWH [3], with a prevalence of approximately 1:30,000 in male neonates [4]. Patients with hemophilia A and B have similar clinical phenotypes, including spontaneous hemorrhages into joints (ankles, knees, and elbows), muscles, or soft tissues [5]. Hemophilia A and B can be divided into three phenotypes on the basis of plasma coagulation factor levels: mild (> 5–40 IU/dL), moderate (1–5 IU/dL), and severe (< 1 IU/dL) [6].

Currently, the developed treatments for hemophilia include prophylactic infusion of coagulation factors and molecular therapies such as antibody, gene, and RNA therapies [7], which significantly prolong the life expectancy and improve the quality of life of PWH. The life span of PWH is close to that of the general population [8]; however, concomitant comorbidities are becoming increasingly prevalent. As a clinically common comorbidity of PWH, osteoporosis is characterized by decreased bone mineral density (BMD) and deteriorated bone microarchitecture, resulting in impaired bone strength and increased risks of fragility fractures [9–11]. A previous study showed that 27% of PWH had osteoporosis, and 43% of PWH had osteopenia [12]. The risk of osteoporotic fractures in PWH was 4.37 times that of the general population of the same sex and age [13]. In PWH, acute massive blood loss during fracture due to coagulation deficiency might obviously increase the risk of osteoporosis and refracture. However, the exact mechanisms underlying hemophilia combined with osteoporosis have not been fully elucidated. Therefore, we review recent progress in research on the pathogenesis and treatments of osteoporosis in PWH.

## Methods

The PubMed, Web of Science, Embase, and Cochrane Library databases were searched up until June 2022 to identify research articles, meta-analyses, and scientific reviews on hemophilia and bone health. The keywords used for literature searches included “osteoporosis,” “osteopenia,” “BMD,” “bone mass,” and “fracture” in combination with “hemophilia” or “factor VIII deficiency” or “factor IX deficiency.” These keywords were combined using the Boolean operators. The search was limited to publications in the English language and those with access to the full text. Articles were carefully studied independently by all authors, and the included articles were agreed upon.

We screened the titles and abstracts to identify relevant articles. The inclusion criteria for the articles were explorations of the pathogenesis of osteoporosis or osteopenia in patients with hemophilia A/B and the treatment of osteoporosis or osteopenia in patients with hemophilia A/B. Literature such as conference abstracts and case reports and articles for which the full text could not be obtained were excluded.

## Results and discussion

### Pathogenesis of osteoporosis in PWH

#### Deficiency of FVIII or FIX

Recent research indicates that FVIII and FIX play critical roles in bone homeostasis. In the intrinsic coagulation pathway, activated FVIII detaches from von Willebrand factor (VWF) to form a complex with activated FIX and then combines with phospholipids on platelet membranes, which in turn activates FX in the presence of Ca^2+^. Activated FX converts prothrombin to thrombin, increases the amount of thrombin, and initiates the process of coagulation [14]. When either FVIII or FIX is deficient, the coagulation cascade cannot be activated appropriately. Interestingly, recent studies found that FVIII knockout (KO) mice had significantly lower hip BMD, cortical bone thickness, and biomechanical strength than their wild-type littermates [15], which indicated that FVIII directly affected bone mass, independent of recurrent hemarthrosis, and differences in physical activity levels. Another study demonstrated that FVIII KO mice had significantly reduced bone mass, cancellous bone fractional area, and trabecular number and increased trabecular separation in the absence of hemorrhage [16]. These findings suggested that the reduction in the BMD of hemophilia A was intrinsic to FVIII deficiency. Moreover, although hemophilia B is less common and milder than hemophilia A, patients with severe hemophilia B had lower lumbar spine and proximal femur BMD than healthy controls [17]. FIX KO mice were also found to have reduced BMD, diminished cortical and cancellous bone mass, impaired bone strength, and increased fracture risk [18]. A study revealed that BMD was substantially increased after FIX replacement treatment, which confirmed that FIX plays a critical role in bone homeostasis [19].

**Fig. 1:**
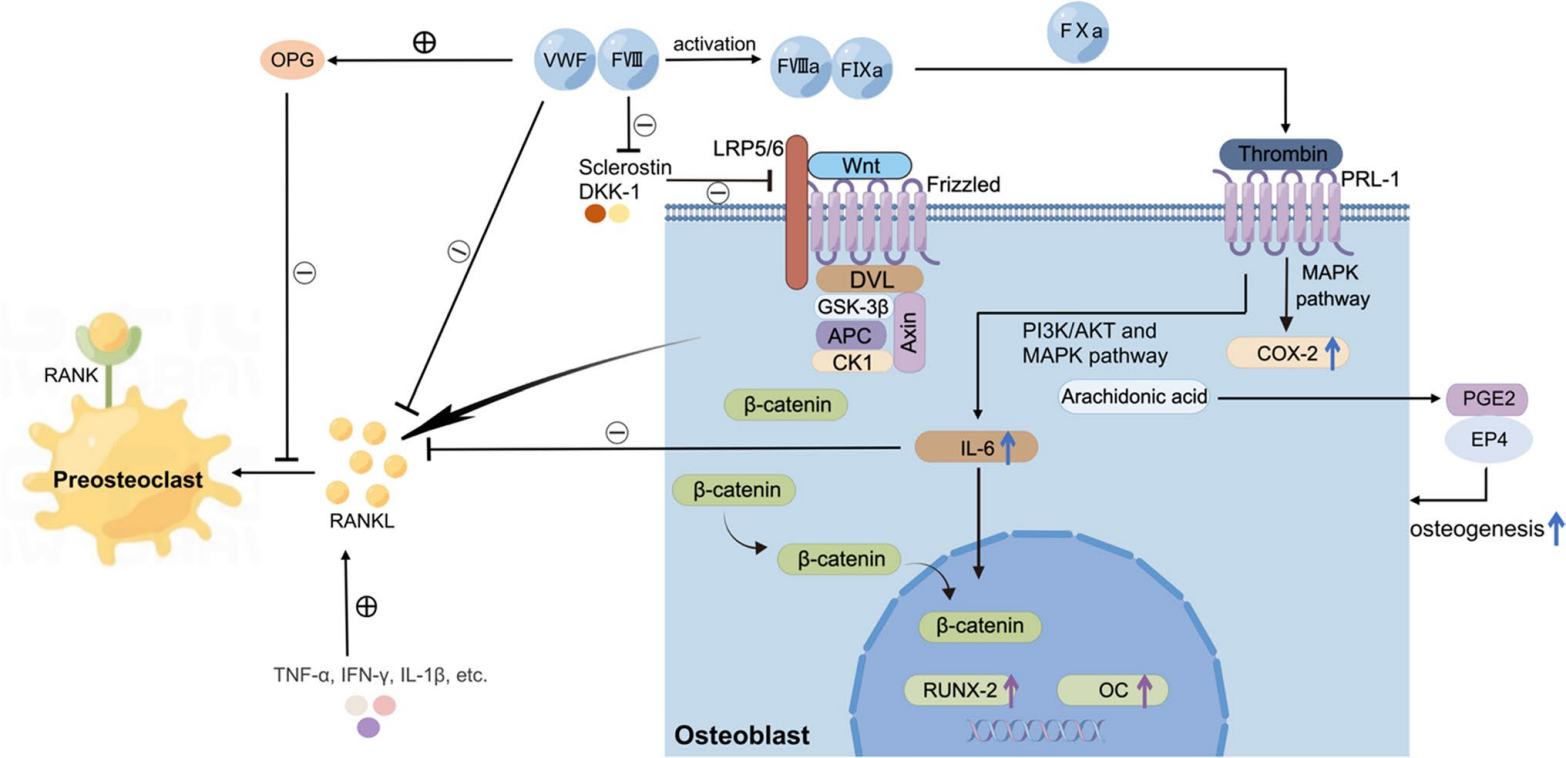
The regulation of FVIII, FIX, thrombin, and cytokines in bone metabolism. FVIII regulates bone homeostasis through the RANK/RANKL/OPG axis. The FVIII/VWF complex binds to RANKL and OPG, inhibits RANKL-induced osteoclastogenesis, and enhances the inhibitory effects of OPG on osteoclasts, thereby promoting osteogenesis. Another possible factor that regulates bone metabolism is thrombin. Activated FVIII detaches from VWF and forms a complex with activated FIX, which further activates FX. FXa catalyzes the conversion of prothrombin into thrombin. Thrombin regulates bone metabolism by binding to PAR-1 on osteoblast membranes, which further upregulates the expression of IL-6. Upregulated IL-6 stimulates the expression of RUNX2 and osteocalcin and reduces the expression of RANKL, further reducing osteoclastogenesis. Cytokines produced from recurrent intra-articular bleeding are also involved in bone metabolism. TNF-α, IFN-γ, IL-1β, etc., directly increase the expression RANKL, resulting in increased bone resorption. On the other hand, FVIII or FIX regulates bone metabolism through the Wnt/β-catenin pathway. FVIII or FIX might decrease the levels of sclerostin, further attenuating the inhibitory effect of sclerostin on the Wnt signaling pathway and thus promoting bone formation. AKT, PI3K-protein kinase B; COX-2, cyclooxygenase 2; EP4, PGE2 receptor 4; IFN-γ, interferon-γ; IL-1β, interleukin-1β; IL-6, interleukin-6; MAPK, mitogen-activated protein kinase; OC, osteocalcin; OPG, osteoprotegerin; PAR-1, protease-activated receptor 1; PGE2, prostaglandin E2; PI3K, phosphoinositide 3-kinase; RANK, receptor activator of nuclear factor-kappa B; RANKL, receptor activator of nuclear factor-kappa B ligand; RUNX2, runt-related transcription factor 2; TNF-α: tumor necrosis factor α

The molecular mechanisms of FVIII or FIX deficiency leading to osteoporosis have been explored. Studies have shown that the RANK/RANKL/OPG signaling pathway plays a role in the development of osteoporosis in PWH [6, 20, 21]. Receptor activator of nuclear factor kappa-B ligand (RANKL) binds to its receptor, RANK, and promotes osteoclastogenesis, resulting in increased bone resorption [22]. Osteoprotegerin (OPG) attenuates osteoclastogenesis by competitive binding with RANKL [23]. This pathway is essential for maintaining bone turnover homeostasis. The FVIII-VWF complex has been found to be directly involved in bone remodeling by binding to RANKL and OPG, inhibiting RANKL-induced osteoclastogenesis, and enhancing the inhibitory effects of OPG on osteoclasts, thereby promoting osteogenesis (Fig. 1) [24]. In FVIII mutant mice, after replacement therapy with FVIII, RANKL expression was reduced by 25% during the differentiation of bone marrow-derived mesenchymal stem cells into osteoblasts [25]. These data suggested that FVIII independently affected bone homeostasis through the RANK/RANKL/OPG pathway. Some clinical studies have found that children and adults with hemophilia had significantly higher RANKL and lower OPG levels than healthy controls [21, 26]. However, other studies have found no significant difference in serum levels of RANKL or OPG or the RANKL/OPG ratio between PWH and healthy controls [27, 28]. These inconsistent results may be related to the complexity of hemophilia and differences in the severity, course, and treatment of hemophilia.

In contrast, few studies have focused on the role of the Wnt/β-catenin pathway in hemophilia. In the Wnt signaling pathway, Wnt binds to a receptor complex on the cell surface that includes Frizzled protein (FZD) and lipoprotein receptor-related protein 5 (LRP5) or LRP6, leading to the recruitment of DVL and the axin–GSK3β–APC–CK1 complex to the receptor. This step suppresses the phosphorylation of β-catenin, leading to increased β-catenin levels in the cytoplasm. The induced β-catenin translocates to the nucleus and activates the transcription of target genes, such as Runx-2 and osteoprotegerin (Fig. 1) [29]. Sclerostin and Dickkopf-1 (DKK-1), antagonists of Wnt, bind to LRP5/6 and inhibit their availability to Wnt ligands [30]. Recent studies found a significantly higher sclerostin level in children with hemophilia than age-matched controls, while no significant difference in serum DKK-1 levels was found between patients and controls [27, 31]. The above results led to the hypothesis that FVIII or FIX deficiency would increase the levels of sclerostin, which would inhibit bone formation through the Wnt signaling pathway and lead to bone loss (Fig. 1). Further studies are needed to explore the role of sclerostin in PWH with osteoporosis.

#### Deficiency of thrombin

Recent studies have shown that FVIII KO and FIX KO mice presented similar bone phenotypes [19, 32, 33], suggesting that bone loss may be attributable to more than just FVIII or FIX deficiency. Recent studies have indicated that the thrombin downstream of FVIII and FVIX may also play a pivotal role in bone homeostasis [6]. This finding suggested that the underlying mechanisms of osteoporosis in PWH were complicated and multifactorial. The process of prothrombin conversion to thrombin is a pivotal step necessary for clot formation. Mutations in the genes encoding FVIII or FIX can result in FX activation disorder and thrombin generation failure. Thrombin has been found to inhibit osteoclast differentiation and stimulate osteoblast proliferation [20], thus promoting bone formation. A study showed that osteoblasts can express thrombin receptors [34], and thrombin plays its role mainly through protease-activated receptors (PARs) and seven-transmembrane domain G protein-coupled receptors [35]. There are two types of thrombin receptors, PAR-1 and PAR-4, expressed on the osteoblast membrane [36, 37]. Thrombin can promote the proliferation of bone marrow mesenchymal stem cells and inhibit the apoptosis of osteoblasts by binding to PAR-1 [37]. PAR-1 KO mice have been shown to exhibit decreased BMD and compromised bone architecture, which was similar to the pattern of FVIII KO mice [35]. The above research indicates that the FVIII/thrombin/PAR1 axis is closely linked to bone remodeling [38].

Moreover, thrombin influences bone metabolism mainly by altering the expression of cytokines [20]. Thrombin is involved in the metabolism of arachidonic acid by binding to PAR-1 on the osteoblast membrane [6]. PAR-1 stimulates the expression of cyclooxygenase-2 (COX-2) through the mitogen-activated protein kinase (MAPK) pathway [39], and upregulated COX-2 catalyzes the conversion of arachidonic acid to prostaglandin E2 (PGE2). PGE2 exerts osteogenic effects by activating PGE2 receptor 4 (EP4) in sensory nerves and inhibiting sympathetic nerve activity in the central nervous system [40]. In addition, activated PAR-1 upregulates the expression of interleukin-6 (IL-6) through the PI3K/AKT and MAPK pathways [6], which stimulates the expression of the pro-osteogenic factors RUNX2 and osteocalcin (OC), promotes the differentiation of mesenchymal stem cells into osteoblasts, and inhibits the apoptosis of osteoblasts (Fig. 1). IL-6 reduces the expression of RANKL (Fig. 1), osteoclastogenic cytokines IL-1, and tumor necrosis factor-α (TNF-α) and stimulates the production of anti-osteoclastogenic cytokines IL-4 and IL-10 [41, 42], which can reduce bone resorption and enhance bone formation. These findings supported the proposition that FVIII or FIX is involved in bone remodeling through thrombin generation and multiple cytokines.

However, the findings of a study indicated that more than 85% of prothrombin knockdown mice did not show a significantly different bone phenotype from wild-type mice [43], which indicated that thrombin deficiency may not be a key mechanism of osteoporosis in PWH. Additionally, a study showed that the immunological profile of untreated PWH presented higher levels of IL-6, IL-4, IL-10, and IL-2 than that of the age-matched healthy controls, which was inconsistent with the above findings [44]. The above studies suggest that the interrelationship between thrombin and bone is quite complicated.

#### Unloading status and immobilization of bone

The unloading status and systemic immobilization of bone can induce disuse osteoporosis [45]. In PWH, long-term spontaneous bleeding in the ankles, knees, or elbows leads to hemarthrosis, cartilage damage, and hemophilic arthropathy [46]. Numerous studies have indicated that BMD is inversely correlated with the severity of arthropathy in PWH [12, 28, 47–49]. The arthropathy severity of PWH was evaluated according to the clinical score of the Orthopedic Advisory Council of the World Federation of Hemophilia [28], and the results showed that the risk of osteoporosis increased by 2.42 times for each 10-point increase in the total clinical score. Moreover, the radiographic joint score could independently explain 23% of the variability in the femoral neck BMD of PWH, and BMD decreased as the arthropathy score increased [50]. The associations between bone turnover markers and hemophilic arthropathy were also explored, and a positive correlation was found between serum sclerostin levels and the severity of joint arthropathy [31]. The levels of the bone resorption markers CTX-1 and NTX-1 were also positively correlated with the degree of arthropathy and number of affected joints [51]. However, another study found no significant correlation between serum levels of RANKL, OPG, and OC and the severity of hemophilic arthropathy [26]. The discrepancies may be attributed to confounders for bone turnover, such as vitamin D nutritional status, physical activity levels, and comorbidities.

The mechanisms of arthropathy associated with low BMD include mechanical unloading and inflammatory stimulation. Hemophilia was found to lead to spontaneous early-onset joint bleeding, which induced joint pain; decreased weight-bearing ability and activity levels; caused impaired muscle and skeletal function and vitamin D deficiency; and increased the risk of osteoporosis, falls, and fractures [52]. In addition, the recurrent intra-articular bleeding and chronic synovitis in PWH also stimulate the production of inflammatory cytokines, such as TNF-α, interferon-γ (IFN-γ), and IL-1 [46, 53, 54], which increases osteoclast activity. In mouse models, the iRhom2/ADAM17/TNF-α pathway was hypothesized to contribute to the activation of osteoclasts and the pathogenesis of osteoporosis in hemophilic arthropathy. This pathway could be prevented by genetic inactivation of TNF-α or iRhom2 or treatment with anti-TNFα biologics [53], which suggested that this signaling axis could be a potential target for the prevention of osteoporosis in arthropathy PWH. In contrast, other study findings indicated that serum levels of IL-1α and IFN-β or TNF-α were significantly lower in PWH than in controls [55, 56]. However, the levels of the inflammatory cytokines IL-1β, IFN-γ, and TNF-α in the synovial fluid of hemophilic mice were significantly increased in injured joints compared with control joints [57]. These study findings suggested that bone turnover in PWH is regulated by mechanical unloading and inflammatory stimulation.

### Progress on the management of osteoporosis in PWH

#### Physical activity and fall prevention

The World Federation of Hemophilia (WFH) guidelines encourage weight-bearing activity for PWH to build bone mass and reduce the risk of osteoporosis, especially for young PWH, as late childhood and adolescence are an important period for the acquisition of peak bone mass. Aerobic exercise, strength/resistance training. and balance and flexibility exercises are suitable for PWH [58]. PWH should be referred to physical therapists for evaluation, education, and instruction before commencing any exercise regimen [59]. Supervised physical therapy is recommended for PWH with arthropathy [59]. A randomized controlled trial (RCT) demonstrated that training in combination with exercise machines (programmed sports therapy) for 6 months had a positive effect on physical performance, especially strength, balance, and endurance, in adult PWH regardless of disease course [60]. Notably, no increased risk of bleeding events was observed in these PWH participating in weight-bearing activity of such an intensity [60].

PWH have a predisposition to falls because of abnormal joint function, impaired mobility, and poor balance [61]. In PWH, the annual incidence of falls is 32–50%, and 53–81% of fractures are due to falls [62]. The WFH guidelines recommend that musculoskeletal assessment should be performed in adult PWH annually and in pediatric PWH every 6 months [59]. A performance-based measure to evaluate functional mobility and fall risk is recommended [63]. When PWH have an increased risk of falling, balance exercises, lower limb strengthening exercises and walking are recommended [64].

#### Supplementation with calcium and vitamin D

In PWH, 47% were found to have vitamin D deficiency, and 25-hydroxyvitamin D levels could independently predict low BMD [48]. The WFH guidelines recommended that PWH receive adequate calcium and vitamin D supplementation [59]. Sufficient calcium intake is necessary for the acquisition of peak bone mass and the maintenance of bone health. Adequate vitamin D intake is helpful to facilitate calcium absorption, improve muscle strength, and reduce the risk of falling [65]. Nevertheless, the efficacy of calcium and vitamin D supplementation for the prevention of osteoporotic fractures is controversial [66]. The large VITAL study showed that daily supplementation with high-dose vitamin D did not improve BMD or bone structure or prevent falls in a generally healthy population [67, 68]. However, the results of the VITAL study cannot be applied to persons with extremely low vitamin D levels or osteoporosis or younger adults since this study did not include any participants with low bone mass or vitamin D insufficiency or young age.

#### Anti-osteoporotic agents

The effective drugs for osteoporosis include antiresorptive, anabolic, and dual-action agents. However, the efficacy of these agents remains undetermined in PWH with osteoporosis. Several studies indicated that the bone resorption marker CTX-1 was significantly increased in PWH [28, 55, 69, 70]. Given the reported higher bone turnover in PWH, antiresorptive therapies such as bisphosphonates or denosumab might be effective in PWH with osteoporosis. However, large-sample prospective drug therapy studies for osteoporosis in PWH are scarce. To date, only one study has evaluated the efficacy of ibandronate for osteoporosis in PWH [62]. In this study, 10 PWH with osteoporosis received ibandronate treatment for 12 months, and the lumbar spine BMD increased by 4.7%, but no significant change was found in the BMD of the total hip or femoral neck [71]. As the sample size of this study was relatively small, larger sample clinical trials are needed.

Denosumab is a fully human monoclonal antibody targeting the bone resorption mediator RANKL that is effective in the treatment of postmenopausal osteoporosis, male osteoporosis, and glucocorticoid-induced osteoporosis [72–74]. Nevertheless, no studies have explored the efficacy of denosumab in PWH with osteoporosis. We have previously treated a man with hemophilia complicated with osteoporotic fracture and delayed healing after surgery. After 4 months of teriparatide treatment and 1 year of denosumab treatment, the fracture healed, and BMD increased significantly. However, the efficacy and safety of denosumab in PWH with osteoporosis need further investigation.

Teriparatide, a recombinant human parathyroid hormone, is an anabolic agent that can significantly increase BMD and reduce the incidence of vertebral fractures [75–77]. The effects of teriparatide on the bones of PWH are unclear. Therefore, studies are needed to assess whether teriparatide is beneficial to PWH with osteoporosis. It is worth noting that PWH are likely to suffer bruising and bleeding from subcutaneous injection of teriparatide, which should be balanced with the effects of teriparatide on bone.

Romosozumab, a humanized monoclonal antibody that binds and inhibits sclerostin, has a unique dual effect of promoting bone formation and inhibiting bone resorption. It is approved by the Food and Drug Administration (FDA) for the treatment of women with postmenopausal osteoporosis and high fracture risk [78]. In studies of children with severe hemophilia A, serum sclerostin levels were significantly increased [27, 31], which suggested that romosozumab might be effective in treating the osteoporosis of PWH. Further prospective clinical studies need to be conducted to determine the efficacy and safety of romosozumab in PWH with osteoporosis.

#### Prophylactic coagulation factor replacement therapy

Prophylactic FVIII:C replacement is a standard treatment for patients with hemophilia A, as it can reduce bleeding episodes, prevent joint damage, and improve quality of life [79]. Prophylactic coagulant factor replacement treatment might be helpful in reducing bone loss in PWH. Direct evidence from animal models showed that FVIII replacement therapy led to a 25% reduction in RANKL levels [25], which suggested that FVIII replacement therapy might be useful in reducing bone loss in PWH. Another mouse study demonstrated that long-term FIX replacement normalized the BMD of FIX KO (FIX^−/−^) mice to that of wild-type mice [19]. Additionally, long-term FVIII replacement was beneficial to preserve the BMD of patients with severe hemophilia [80]. Furthermore, prophylactic coagulation factor replacement therapy also reduces the occurrence of bleeding, facilitates joint mobility, and thus reduces the risk of disuse osteoporosis. Nonetheless, the generation of autoantibodies to FVIII or FIX, such as anti-FVIII or FIX alloantibodies, is a major complication of at least one-third of patients with severe hemophilia A and approximately 3 to 5% of those with severe hemophilia B [6, 81] that leads to PWH unresponsiveness to replacement therapy. To overcome this limitation, the FDA approved new nonreplacement therapeutic strategies, such as enhancing coagulation and inhibiting anticoagulant pathways [81, 82]. However, less is known about the effects of these new agents on bone homeostasis.

In summary, appropriate weight-bearing exercise and adequate vitamin D and calcium intake are recommended for PWH to prevent osteoporosis. Currently, limited data are available on the efficacy of anti-osteoporotic treatment in PWH. Since osteoporosis/osteopenia becomes an increasingly pronounced problem as PWH age, determining the effectiveness of anti-osteoporotic treatment in PWH is of great clinical value.

## Conclusion and insights

PWH usually have a high risk of osteoporosis and fractures, which significantly increase morbidity and mortality. Several multicenter studies have evaluated bone status in PWH [83, 84]; however, these real-world studies are inadequate. The mechanism of occurrence of osteoporosis in hemophilia is complicated and multifactorial, including an intrinsic FVIII or FIX deficiency, an impaired thrombin/PAR1 pathway, inadequate weight-bearing activity levels, and inflammatory stimulation. Exercise and physical activity, fall prevention, and adequate supplementation with calcium and vitamin D are recommended for PWH. Prophylactic coagulation factor replacement treatment is beneficial to reduce bone loss in PWH with osteoporosis. However, studies on the effects of anti-osteoporotic agents on the bones of PWH with osteoporosis are extremely rare, and further RCTs are necessary to clarify the efficacy and safety of anti-osteoporotic agents in PWH.
